# Latent Tuberculosis Infection Diagnosis among Household Contacts in a High Tuberculosis-Burden Area: a Comparison between Transcript Signature and Interferon Gamma Release Assay

**DOI:** 10.1128/spectrum.02445-21

**Published:** 2022-04-13

**Authors:** Sheetal Kaul, Vivek Nair, Shweta Birla, Shikha Dhawan, Sumit Rathore, Vishal Khanna, Sheelu Lohiya, Shakir Ali, Shamim Mannan, Kirankumar Rade, Pawan Malhotra, Dinesh Gupta, Ashwani Khanna, Asif Mohmmed

**Affiliations:** a International Centre for Genetic Engineering and Biotechnology, New Delhi, India; b Department of Biochemistry, School of Chemical and Life Sciences, Jamia Hamdard, New Delhi, India; c Partasia Biopharm, New Delhi, India; d Society for Health Allied Research & Education (SHARE INDIA), New Delhi, India; e All India Institute of Medical Sciences, New Delhi, India; f Chest Clinic (Tuberculosis), Lok Nayak Hospital, New Delhi, India; g WHO-India Country Office, New Delhi, India; Johns Hopkins University School of Medicine

**Keywords:** latent tuberculosis infection, transcript signature, biomarker, interferon gamma release assay, household contacts, machine learning

## Abstract

Diagnosis of latent tuberculosis infection (LTBI) using biomarkers in order to identify the risk of progressing to active TB and therefore predicting a preventive therapy has been the main bottleneck in eradication of tuberculosis. We compared two assays for the diagnosis of LTBI: transcript signatures and interferon gamma release assay (IGRA), among household contacts (HHCs) in a high tuberculosis-burden population. HHCs of active TB cases were recruited for our study; these were confirmed to be clinically negative for active TB disease. Eighty HHCs were screened by IGRA using QuantiFERON-TB Gold Plus (QFT-Plus) to identify LTBI and uninfected cohorts; further, quantitative levels of transcript for selected six genes (*TNFRSF10C*, *ASUN*, *NEMF*, *FCGR1B*, *GBP1*, and *GBP5*) were determined. Machine learning (ML) was used to construct models of different gene combinations, with a view to identify hidden but significant underlying patterns of their transcript levels. Forty-three HHCs were found to be IGRA positive (LTBI) and thirty-seven were IGRA negative (uninfected). *FCGR1B*, *GBP1*, and *GBP5* transcripts differentiated LTBI from uninfected among HHCs using Livak method. ML and ROC (Receiver Operator Characteristic) analysis validated this transcript signature to have a specificity of 72.7%. In this study, we compared a quantitative transcript signature with IGRA to assess the diagnostic ability of the two, for detection of LTBI cases among HHCs of a high-TB burden population; we concluded that a three gene (*FCGR1B, GBP1*, and *GBP5*) transcript signature can be used as a biomarker for rapid screening.

**IMPORTANCE** The study compares potential of transcript signature and IGRA to diagnose LTBI. It is first of its kind study to screen household contacts (HHCs) in high TB burden area of India. A transcript signature (*FCGR1B, GBP1*, & *GBP5*) is identified as potential biomarker for LTBI. These results can lead to development of point-of-care (POC) like device for LTBI screening in a high TB burdened area.

## INTRODUCTION

Latent tuberculosis infection (LTBI) is a state of persistent immune response to stimulation by Mycobacterium tuberculosis antigens without any clinical manifestation of active tuberculosis (TB) (1). The global prevalence of LTBI is estimated to be at ∼33% (2); though it is a non-communicable asymptomatic condition, but LTBI individuals stand at a significant risk of progression to active TB due to various factors such as low immunity, co-morbidities etc.

Identification of novel biomarkers for rapid diagnosis of LTBI is a prerequisite to eradicate TB since the current diagnostic methods for detection of LTBI are either subjective or expensive. Several studies have shown variations in transcription levels between healthy controls versus active TB and LTBI versus active TB populations (3–5). However, not many studies have identified transcript signatures that can distinguish LTBI individuals from uninfected, within the household contacts (HHCs) of active TB population. Such signatures can be useful for rapid screening of the already at risk-HHCs and thus could be an important tool in achieving the goal of End-TB.

Machine learning techniques are being used as a tool to identify hidden but significant underlying patterns among complex data sets. One such machine learning unsupervised approach is cluster analysis that is a collection of methods for defining subgroups of individuals with high heterogeneity (6–8). It is a widely used exploratory approach in biological studies discovering substructures inherent in a given data set. It is a hypothesis generating approach that assigns patients to clusters based on certain characteristics, so that homogeneity is high within the cluster and at the same time low between-group (9).

In the present study, for segregating cohorts of LTBI and uninfected individuals, an interferon gamma release assay (IGRA) based screening of HHCs - parent, sibling, spouse, child and others, of index TB cases was carried out. The transcript levels of six genes, that previously showed an association with TB-infection, namely, *TNFRSF10C* (Tumor Necrosis Factor Receptor Super Family Member 10c) (10)*, ASUN* (Asunder spermatogenesis regulator)*, NEMF* (Nuclear Export Mediator Factor) (11), *FCGR1B* (Fc gamma receptor 1B)*, GBP1* (Guanylate binding protein 1) and *GBP5* (Guanylate binding protein 5) (12), were quantified by qRT-PCR.

For comprehensive analysis, two approaches of machine learning (ML) were applied. The outcomes of the ML approaches were further validated using various statistical analyses. This study is first of its kind to assess a transcript signature of three genes and compares it to IGRA for efficacy to diagnose LTBI among HHCs of high TB burdened Indian population, which paves a way for a broader trial and validation of this marker. A three-gene (*FCGR1B, GBP1, and GBP5*) transcript signature is identified which can be used as a biomarker for rapid screening of HHCs to diagnose LTBI.

## RESULTS

### Selection of LTBI and uninfected cohorts within household contacts of index TB populations.

**FIG 1:**
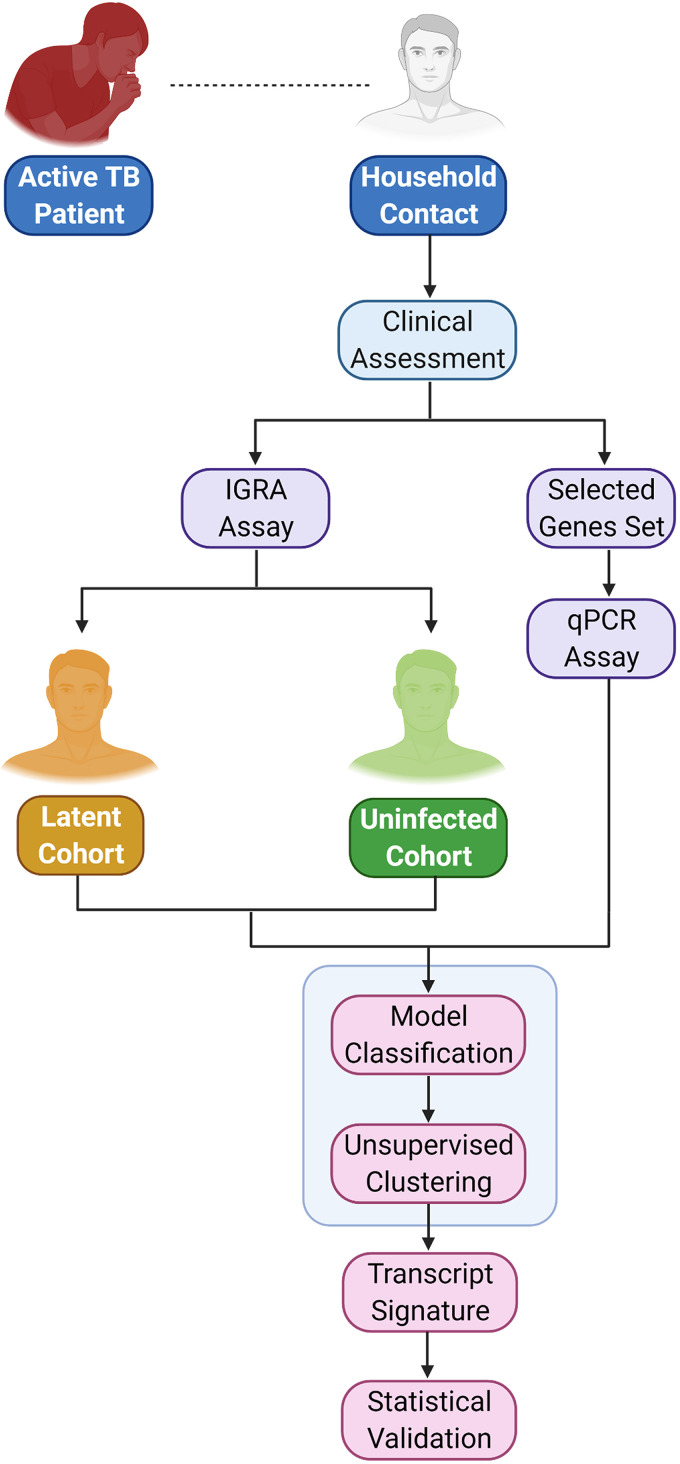
Flow chart showing the experimental design of the study.

Eighty HHCs of confirmed pulmonary TB Indian patients were screened by IGRA to segregate LTBI and uninfected individuals. IGRA assay was carried out twice using the same sample (supernatant from 4 tubes- Nil, TB1, TB2 and Mitogen) for each of the participant. Only those samples which showed repeated results in this assay, were considered in the study (Fig. 1). In case of any discrepancies between the technical repeats, the participant was eliminated from the study. Among 80 HHCs, 54% (*n *= 43) tested IGRA positive, and 46% (*n *= 37) tested IGRA negative; these cohorts were identified as LTBI and uninfected respectively (Table 1).

**TABLE 1:** Clinical and demographic characteristics of enrolled household contacts^*a*^

	Household Contacts		
Characteristic	IGRA +ve	IGRA -ve	Total
*N* (%)	43	37	80
Age median (IQR)	30 (14–58)	27 (15–63)	29 (14–63)
Gender			
Male *N* (%)	22 (53)	20 (47)	42
Female *N* (%)	21 (55)	17 (45)	38
Index Cases			
Cat I contacts (%)	36 (56)	28 (44)	64
Cat II contacts (%)	2 (22)	7 (78)	9
Cat IV contacts (%)	5 (71)	2 (29)	7
Diabetes			
Yes	3	1	
No	40	36	
Smoking			
Yes	2	3	
No	41	34	
Alcoholic			
Yes	3	4	
No	40	33	
Diet			
Veg	14	17	
Non-veg	25	15	
Ventilation			
Good	4	4	
Avg	19	17	
Poor	20	16	

a IGRA: interferon gamma release assay; IQR: interquartile range. Veg.: vegetarian. Cat I: Drug Susceptible TB; Cat II: Drug Susceptible-Relapse; Cat IV: Drug Resistant TB. Ventilation: No. of rooms/No. of members (<0.5: Poor; 0.5–0.75: Average; >0.75 Good).

**FIG 2:**
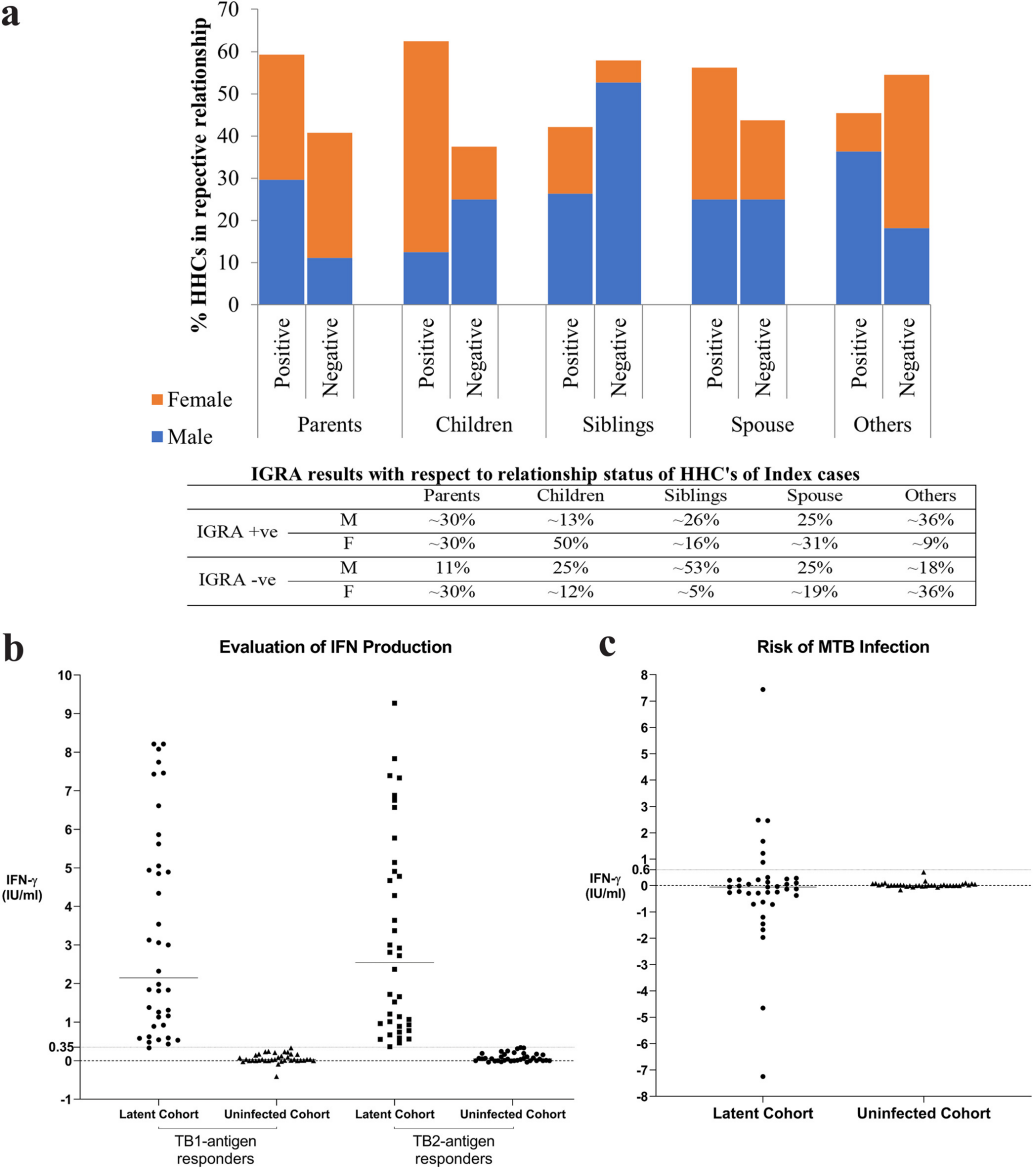
a: This graph shows LTBI predominance in children (relationship of study participant with respect to index TB cases) out of which females tend to have a higher percentage compared to males. b: The IFN-γ T-cell response was evaluated in LTBI and uninfected cohort of HHCs. Horizontal lines indicate the median whereas the dotted line represents the cutoff value of 0.35 IU/mL which decides the IGRA status of the individual. c: Observed differences between TB1 and TB2 values (TB-TB1), stratified by risk of LTBI (*n* = 43) and uninfected (*n* = 37) cohorts of HHCs. Subjects with values for TB1 or TB2 outside the linear range of the assay (>10.0 IU/mL) were excluded. Horizontal lines indicate the median. The individuals who are at a higher risk of progression to active TB are represented by the dots above the cutoff value of 0.6 IU/mL.

No statistical significance could be seen for the various demographic characteristics between the two cohorts. We further analyzed the relationship status of the HHCs with respect to their IGRA results and gender-wise distribution to assess any correlation; percentage wise distribution among different relationship categories for males: females in the LTBI and uninfected cohort (Fig. 2a). No statistical significance was found for age and gender between latent and uninfected cohorts (Table S2 [File 01] in the supplemental material).

To evaluate the level of IFN-γ production in IGRAs, levels of antigen-specific CD4^+^ and CD8^+^ T cells in LTBI cohort and uninfected cohort (Fig. 2b) were compared. To assess the risk of M. tuberculosis infection, difference of TB1 from TB2 was analyzed. A higher TB2 antigen response (TB2-TB1 > 0.6 IU/mL) was observed in six (∼14%) of LTBI subjects. Samples with values for TB1 or TB2 > 10.0 IU/mL were excluded since they were outside the linear range of the assay (Fig. 2c).

### Variation in transcription levels of selected genes between LTBI and uninfected cohorts.

**FIG 3:**
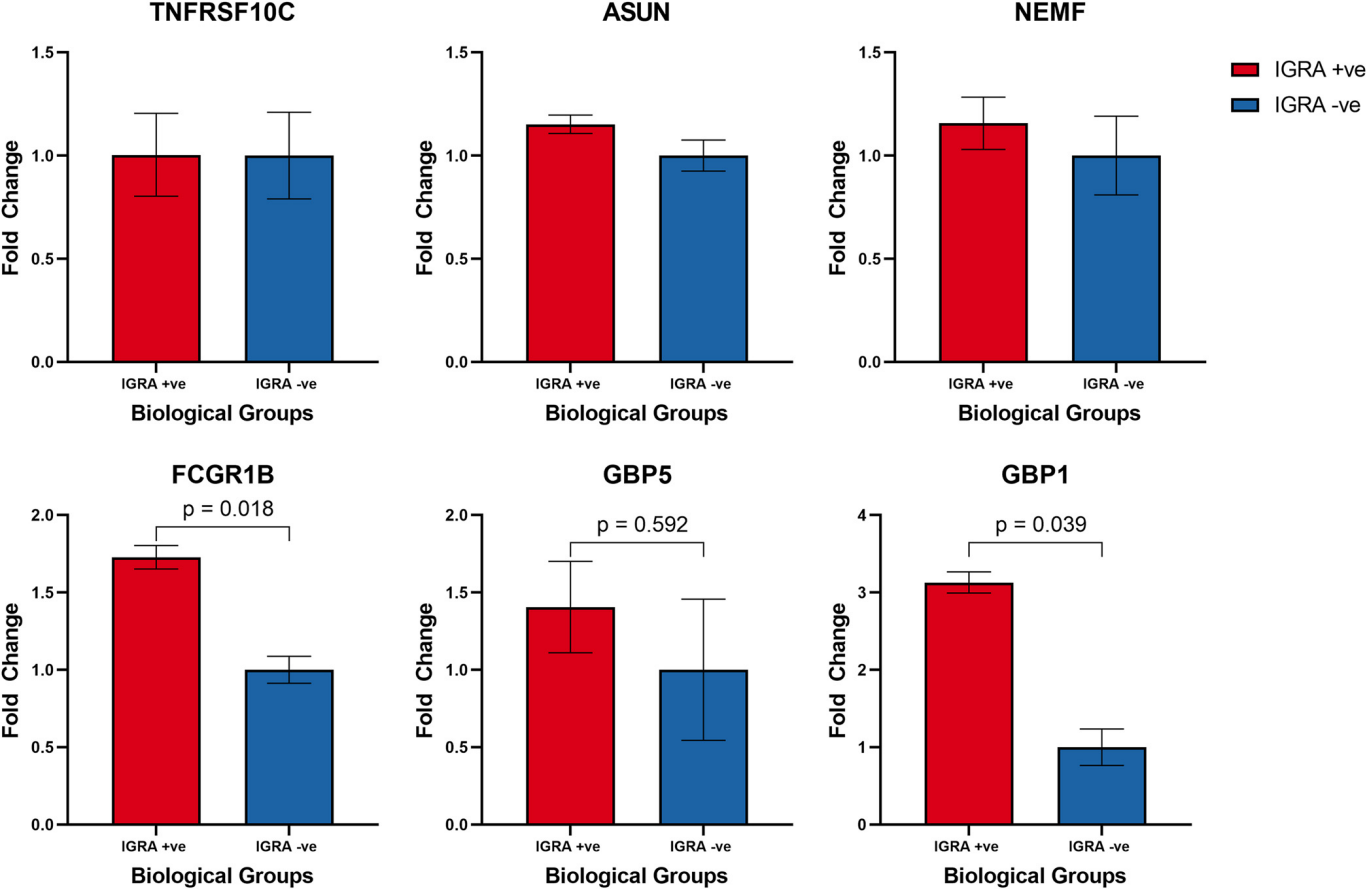
Graphical representation of average relative expression of *TNFRSF10C, ASUN, NEMF, FCGR1B, GBP5*, and *GBP1* as estimated by quantitative RT-PCR (vertical bars represent SEM). Mann-Whitney U test was used to compare the differences among the groups.

Next, the relative transcription levels of six prioritized genes, namely, *TNFRSF10C*, *ASUN*, *NEMF*, *FCGR1B*, *GBP1*, and *GBP5,* that previously showed an association with M. tuberculosis infection in different geographical locations, was assessed (10–12). qRT-PCR based analysis was carried out to estimate transcript levels of these genes among 80 HHCs, with a view to evaluate the potential of these genes to differentiate LTBI and uninfected cohorts among HHCs. *FCGR1B* gene presented the most significant differential expression, as the relative fold expression in the LTBI cohort was significantly higher compared to uninfected cohort (*p*0.018) followed by *GBP1* gene (*p*0.039) and *GBP5* gene (*p*0.592) (Fig. 3). Relatively low level of differential expression could be seen for *ASUN* and *NEMF* genes, which was not significant. However, no significant difference was observed for *TNFRSF10C* gene.

### Unsupervised cluster analysis.

**FIG 4:**
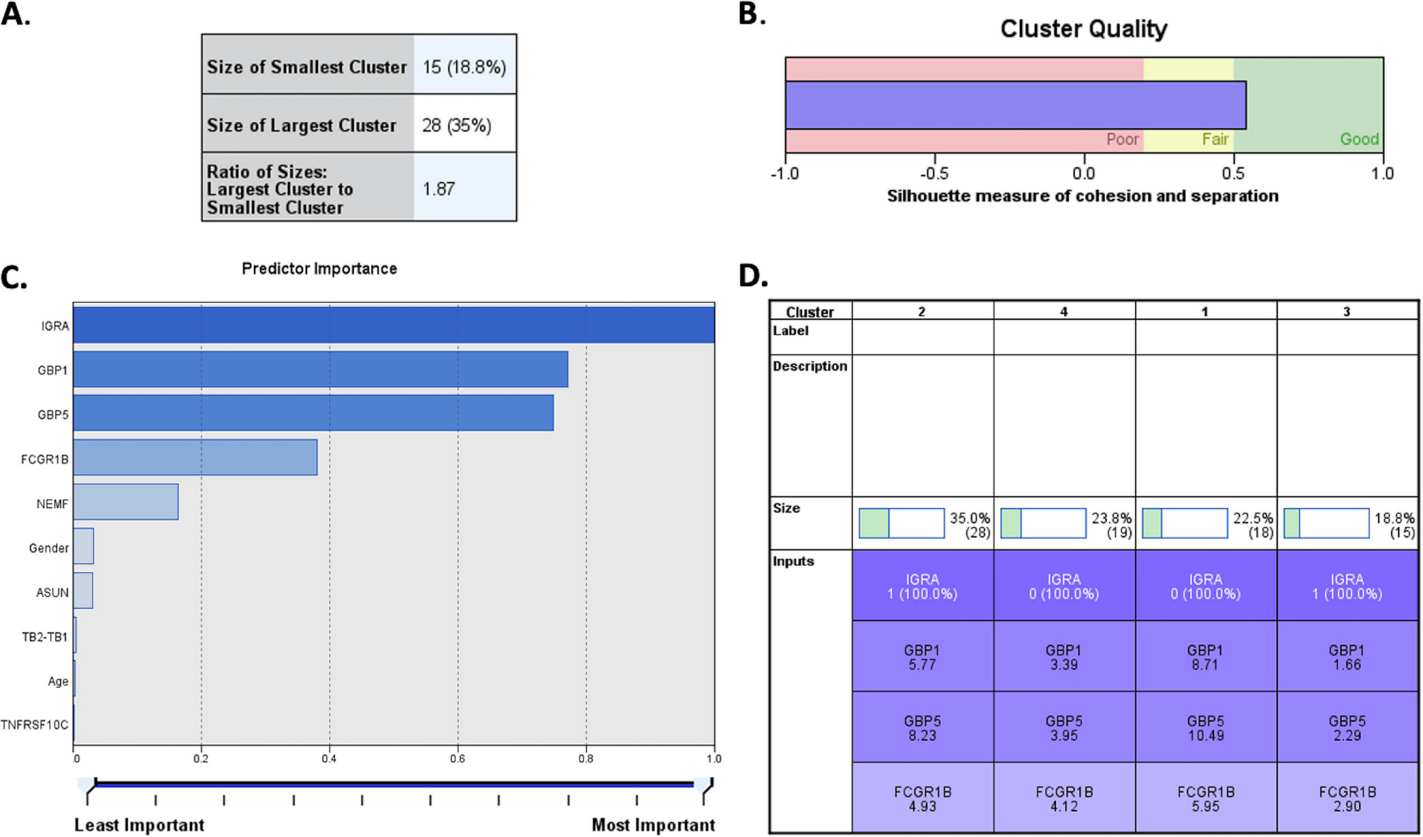
Unsupervised cluster analysis based upon immunological parameters (IGRA positive/negative status, TB1, TB2 values), expression data of the six prioritized genes (*TNFRSF10C, ASUN, NEMF, FCGR1B, GBP1*, and *GBP5*), and epidemiology details (age and gender). (A) Ratio of largest/smallest cluster formed was <2.0. (B) Values of silhouette measures of cohesion and separation off the model was found to be >0.5. (C) Predictor importance of all the parameter analyzed; four parameters showing highest predictor importance were used in building the model. (D) Four clusters identified using these parameters, their sizes and average values of each of parameter for the HHCs in respective cluster.

Unsupervised clustering technique was implemented to find any hidden pattern across the recruited HHCs of index TB cases, in order to group the participants in separate clusters based on their intra-cluster’s similarities and inter-cluster’s differences. A best cluster model was identified that had four groups that were found to be different from each other based on IGRA status (positive/negative) and expression levels of the three genes namely, *FCGR1B*, *GBP1*, and *GBP5*. The cluster model has an acceptable ratio of 1.87 and the silhouette measure of cohesion and separation for our cluster analysis was found to be more than 0.5 indicating it to be an acceptable robust cluster (Fig. 4) and suggesting that the within-cluster distance and the between cluster distance was significant.

### Classification model for IGRA class-prediction.

The distribution of other available parameters, which were not used in the formation of the final cluster model, were assessed (Table 2).

**TABLE 2:** Characteristic features of distribution of samples among different clusters identified through unsupervised cluster analysis based upon expression data of six prioritized genes and other epidemiology details

Features in clusters	Cluster-1	Cluster-2	Cluster-3	Cluster-4
Sample no., *n* (%)	18 (22)	19 (23)	15 (18)	28 (35)
IGRA	Negative	Negative	Positive	Positive
Expression level of *FCGR1B* (avg ΔCt)	5.95 (Lowest exp)	4.12	2.90 (Highest exp)	4.93
Expression level of *GBP1* (avg ΔCt)	8.71 (Lowest exp)	3.39	1.66 (Highest exp)	5.77
Expression level of *GBP5* (avg ΔCt)	10.49 (Lowest exp)	3.95	2.29 (Highest exp)	8.23
Female:Male	7:11 (0.63)	10:9 (1.11)	10:5 (2.0)	11:17 (0.64)
TB2-TB1	–0.17 to 0.1	–0.17 to 0.51	–7.25 to 7.44	–4.65 to 2.48
Age (range)	15-63	15-50	16-53	14-58
Age (mode)	15	25	35	40
Age (avg)	33.44	32.6	31.4	32.14
Age (median)	32.5	27	33	29.5

The classification models were developed using expression data of the six prioritized genes, age and gender as input parameters. Out of the several generated models, based on all the algorithms, present in the WEKA package, the best three models were selected. Based on the feature selection technique, we concluded that the expression values of the three genes: *FCGR1B*, *GBP1*, and *GBP5* could differentiate and predict the outcome of IGRA analysis of the subject with an accuracy of 67.9% (Table 3). The association of the three genes was further validated through statistical analysis.

**TABLE 3:** Expression signatures identified using feature selection technique for predicting LTBI or uninfected sample

Inputs	Algorithm	Sensitivity	Specificity	Accuracy
*FCGR1B, GBP1, GBP5*	Rules.OneR	74.4	60.55	67.9
*GBP1, GBP5*	Rules.OneR	74.4	60.5	67.9
*NEMF, GBP1, GBP5, FCGR1B*	Rules.OneR	74.4	60	67.9

### ROC analysis.

**FIG 5:**
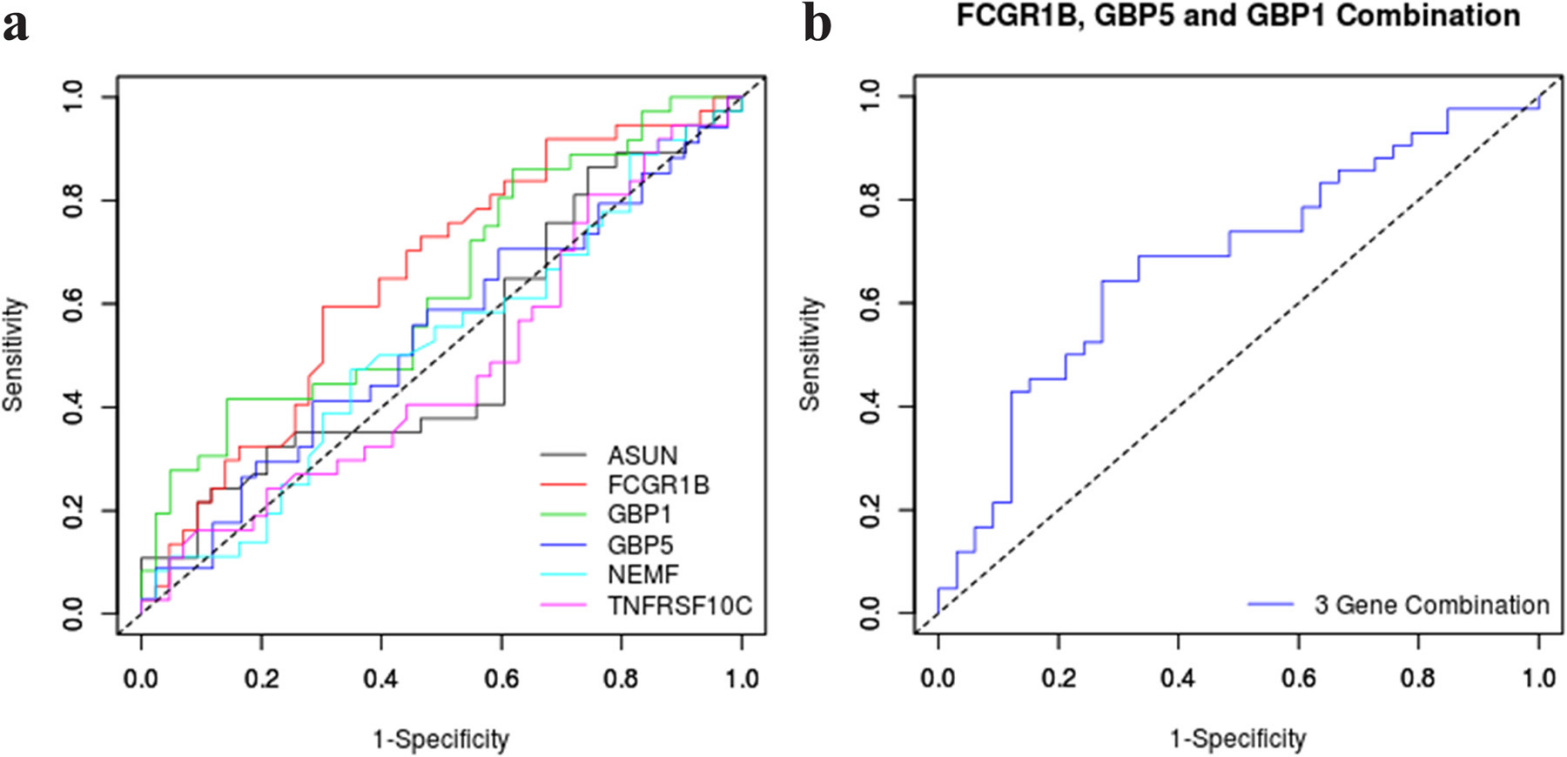
a: ROC analysis of expression patterns of individual six genes to assess their potential to discriminate LTBI and uninfected cohorts. b: ROC analysis for the combination of expression pattern of selected genes (*FCGR1B, GBP1*, and *GBP5*) to assess their potential to discriminate LTBI and uninfected cohorts.

ROC analysis was performed on Ct difference to *GAPDH* for all the genes (Fig. 5a). Further, to validate the association of the three genes as a combination among these six genes, binomial logistic regression and ROC was performed to ascertain the gene combinations to correctly classify the study subjects. The discrimination of LTBI and uninfected cohorts using the combination of *FCGR1B*, *GBP1*, and *GBP5* genes achieved the highest area under the curve (AUC) of 0.68 (95% CI = 0.56-0.80). The threshold for this 3-gene combination that discriminated the IGRA positive groups from IGRA negative groups with the greatest accuracy was identified using the Youden index. This threshold achieved a sensitivity of 0.64 (95% CI 0.48–0.78) and a specificity of 0.72 (95% CI 0.55–0.87), giving a positive likelihood ratio of 2.36 (Fig. 5b). ROC graphs for other gene combinations have been shown in Fig. S1 in the supplemental material.

## DISCUSSION

Transcript signatures can play an important role in differentiating the healthy population from latent individuals as well as in predicting the progression from LTBI to active TB disease. These signatures have emerged ahead of proteomics and metabolomics biomarkers for diagnosis of TB (13–16); this strategy takes advantage of well-established sample-processing pathways as well as rapid multiplex PCR platforms (17, 18). Though, IGRA can be used for the screening of latent infected individuals, however, technical difficulties including collection of larger volume of blood, limited reproducibility of the assay, as well as the tedious sample processing limits its usage in large numbers. Therefore, in the present study, our goal was to assess the levels of transcript signatures to distinguish LTBI and uninfected cohorts among the HHCs of index TB cases within an Indian geographical and ethnic population.

A number of transcript signature markers have been reported to differentiate active TB cases across different geographical areas around the world. Maertzdorf et al. earlier reported that transcript levels for *FCGR1B*, *GBP1*, and *GBP5* could discriminate between active TB versus LTBI groups and active TB versus healthy control groups using South African population as study group (12). Similarly, Lee et al. reported *ASUN* and *NEMF* transcript levels could differentiate LTBI from healthy individuals in a cohort from Taiwan (11). Lastly, Wang et al. showed in a study group from Fudan that *TNFRSF10C* could distinguish between individuals in the active TB, LTBI and healthy control groups using transcriptional profiling study (10). Roe et al. identified a transcript signature of three genes with a high predictive value to identify progression of TB disease in HIV patients in 3 months which shows the importance of a transcript signature-based biomarker (19).

In the present study, we evaluated the potential of these six transcript signatures for defining LTBI cases in a high TB burden Indian population by qRT-PCR since the identification and treatment of LTBI cases form an essential component of the TB eradication programs. The expression of *FCGR1B* and *GBP1* was found to be significantly higher in the LTBI cohort compared to the uninfected cohort, followed by *GBP5*, however *GBP5* differential expression was not statistically significant. The present results were consistent with the previous study by Maertzdorf et al. on the usefulness of expression profile of *FCGR1B*, *GBP1*, and *GBP5*. No significant difference in the transcript levels for the genes *TNFRSF10C*, *ASUN*, and *NEMF* was seen in the present study.

The genes *FCGR1B*, *GBP1*, and *GBP5* play a role in the host immune response during mycobacterial infection. Satproedprai et al. reported that upon bacterial infection, *FCGR1B* upregulation induced humoral immune response and played a role in lung inflammation (20). It was also reported as one of the most differentially expressed genes in individuals with TB and LTBI than uninfected individuals (12). Likewise, *GBP1*and *GBP5* belong to a family of IFN-γ-inducible Guanylate binding proteins (GBPs) and play a key role in host response to intracellular infection (21–23).

ML based cluster analysis was utilized with a view to decipher any correlation among all possible combinations of parameters in LTBI and uninfected cohorts of HHCs. Based upon this analysis, four significant distinguishable clusters were identified; the LTBI cases were distributed into two distinct clusters (cluster 3 and 4) which showed variation for the parameter: IFN-γ. In the LTBI cohort, a higher TB2 antigen response (TB2-TB1 > 0.6 IU/mL) was observed in six subjects (∼14%), suggesting that these subjects are at a higher risk of progression to active TB. The IFN-γ response from CD8^+^ T cells elicited by MTB complex-specific antigens ESAT-6 and CFP-10 are more frequently detected in subjects with active TB than LTBI (24). Therefore, a higher TB2 antigen response elicited by CD8^+^ T cells is said to be associated with severe MTB infection and, consequently, with increased risk of progression to active TB (25). In an earlier study by Petruccioli et al. and Barcellini et al., a higher TB2 antigen response was reported in a subgroup of LTBI contacts with a higher TB burden (26). The cluster 4 showed higher IFN-γ values in TB1 and TB2 compared to cluster 3, which can therefore be said to have a higher TB burden. Therefore, such clustering could help in targeting individuals with LTBI who are at a higher risk of progression to active TB and could also help in solving the dilemma in clinical practice of targeting preventive treatments to groups which are at a higher risk of progression to active TB. The WHO recommends 6–9 months of daily isoniazid preventive therapy (IPT) or a 3-month of weekly rifapentine plus isoniazid (3HP) or a 3-month regimen of daily isoniazid plus rifampicin for the preventive treatment of LTBI (27). The clinical implications of identifying a transcript signature could be towards mass screening of contacts in endemic populations to identify LTBI for targeted preventive therapy. CORTIS trial by Scriba et al. showed the utility of RISK11 signature to predict progression of incident tuberculosis in exposed populations; however, the 3HP based therapy in RISK11 positive group was not successful in preventing the disease based on population (28). In contrast to RISK11, the gene signature screened in this study are for identifying LTBI, and thus the implication could be preventive therapy to contacts having LTBI rather than specifically targeting high risk individuals.

Model classification was used to assess the diagnostic performance of the exact combination of genes and indicated the combination of *FCGR1B*, *GBP1*, and *GBP5* genes to have maximum predictive power in differentiating the LTBI cohort from the uninfected cohort. Further, ROC analysis of expression levels of these combinations confirmed the potential of the 3 gene expression signature. Few limitations of the study are low sample size, which may explain the low accuracy of the transcript signature and absence of an independent, external validation data set. This was not a longitudinal study and could not develop any correlate with transcriptomic biomarker and disease progression. Regardless, we showed the discriminative power of the transcript signature in our population, which was specific for Indian population and hence a different geographical and ethnic cohort when compared with other biomarker studies performed in the past. Therefore, further studies utilizing a larger sample size are required to identify differentially expressing genes to discriminate LTBI from uninfected individuals. Additional studies are needed to be carried out to examine the exposed but uninfected group of HHCs that are immunologically and genetically unique.

In conclusion, this study is not an unbiased analysis of gene expression but rather the validation of previously published genes associated with TB infection in other populations. This was performed using the published signatures to screen a highly exposed Indian population with a view to assess the potential of the targeted transcriptomic markers and their correlation with the IGRA results. Since these gene signatures may not necessarily be generalizable to geographically, epidemiologically and ethnically diverse populations, therefore identifying a distinct transcript signature to diagnose both LTBI and predict disease progression, which can be used globally, remains challenging.

## MATERIALS AND METHODS

### Ethics statement.

The study protocols were approved by institutional ethics committee and informed consents were obtained from all the study participants.

### Study participants and sample collection.

A total of 80 HHCs of bacteriologically confirmed active pulmonary TB patients (diagnosed either as drug susceptible TB [Cat I], drug susceptible-relapse [Cat II] or Drug resistant TB [Cat IV] cases as per National Guidelines at Lok Nayak Chest Clinic, National TB Elimination Program-New Delhi, Government of India), who were in close contact of more than or equal to 8 h/day for at least 3 months, with the respective index TB patient after onset of the infection, were recruited for the study (Fig. 1). Sample size was estimated based on statistical power of 0.8, medium effect size of 0.6 and significance criterion α = 0.05 using the R package pwr (29, 30). The medium effect size of 0.6 was considered in the case of HHCs as they were at a higher probability of having LTBI than the general population (31). Further, all the HHCs were screened for TB-disease using chest radiography (CXR) and GeneXpert MTB Rif testing (Cepheid, USA), and only those found to be negative for TB-disease, and having no previous TB-history, were recruited for the study. The exclusion criteria for the recruited HHCs was that they should not have any history of diabetes, smoking and alcohol use. 4 mL of venous blood was collected from the subjects in Lithium Heparin tubes (367886, BD, UK) for IGRA analysis and 500 μL was collected in K2E tubes for RNA extraction (367836, BD, UK).

### Interferon gamma release assay (IGRA) (QuantiFERON-TB Gold Plus [QFT-Plus]).

IGRA was performed using QFT-Plus kit (Qiagen, Germany) for all the HHCs as per manufacturer’s protocol (622180, Qiagen, Germany) and the test results were interpreted using QuantiFERON-TB Gold Plus Analysis Software.

### RNA extraction and cDNA synthesis.

Total RNA was extracted from 200 μL whole blood using a RNeasy mini spin column (74104, Qiagen, Germany) following manufacturer’s instructions. An amount of 100 ng of RNA was used for each cDNA synthesis reaction using reverse High-Capacity cDNA RT-kit (4368814, ThermoFisher SCIENTIFIC, USA) following manufacturer’s instructions.

### Quantitative Real time-PCR (qRT-PCR).

Relative transcript levels of 6-genes (*TNFRSF10C*, *ASUN*, *NEMF*, *FCGR1B*, *GBP1*, and *GBP5*) were measured by qRT-PCR, which was performed in triplicates. Primer sequences for each gene are listed in Table S1 (File 01) in the supplemental material. The relative amount of expressed RNA was calculated by comparing it with the expression of the housekeeping gene *GAPDH* (Glyceraldehyde-3-Phosphate Dehydrogenase) using the 2^−ΔΔCT^ (Livak) method.

### Machine Learning based approaches.

**(i) Cluster analysis.** Classifying the study cohort into distinct clusters was done based on 10 parameters including expression data of the six selected genes, immunological parameters from IGRA assays (TB1-TB2 values, IGRA positive/negative) and epidemiological data like age and gender. Two-step cluster analysis (SPSS 26.0, IBM Corporation, USA) was performed for clustering where both categorical and continuous variables were used as input parameters. The analysis involved two stages which resulted in a large number of clusters; filtered down to the best numbers using Schwarz's Bayesian Information Criterion. Briefly, in the first stage a cluster feature tree was constructed and the cases were grouped into these preclusters followed by implementation of the hierarchical clustering algorithm on the preclusters.

In a preliminary evaluation using cluster analysis, three main approaches were used for defining the study cohort. The first approach involved grouping the participants based on demographic details and sought to describe age and gender differences among the groups. In the second approach, expression data of the six genes along with immunological data, were used to cluster the participants in different groups followed by the third approach where all the data (demographic, expression and immunological) were used. Of the total 73 clusters, only those clusters in which the input parameters indicated significant differences, were included.

**(ii) Classification model for IGRA class-prediction.** WEKA (32) was used to perform a class prediction study to predict whether the participant was IGRA positive/IGRA negative based on the expression data of the six prioritized genes, age and gender. The training set was used to evaluate the performance of 120 algorithms from eight main classifiers (bayes, functions, lazy, meta, mi, misc, rules, trees) available in WEKA (v3.8.4). Various combinations of different input parameters were evaluated and the algorithm with the best performance in the leave-one-out cross-validation (LOOCV) was selected and a classification model was built to differentiate between the IGRA positive (43) and IGRA negative (37) individuals. Due to limited number of participants, it was not possible to prepare separate training and validation data sets. Therefore, the LOOCV technique was used to utilize the available information optimally. In the LOOCV technique, the models are trained and validated so that each record is used for training and internal testing. The LOOCV technique has widely been used to solve several classification problems where data is scarce (33, 34). Further, all the feature selection techniques (WEKA) were used to identify the most significant and discriminatory input parameters leading to the best classification model.

### Statistical validation.

Differences between the IGRA positive and IGRA negative cohorts were evaluated by Mann-Whitney U-test in SPSS 26.0 (IBM Corporation, USA). The diagnostic ability of the selected genes to discriminate between the study groups was evaluated by performing receiver operating characteristic (ROC) analysis for the genes individually and the overall accuracy was assessed by area under curve (AUC) values in easyROC web-tool (35). Binary logistic regression analysis was then used to examine combinations of genes followed by ROC analysis to validate the model.
