# Supplementary material for: Latent Tuberculosis Infection Diagnosis among Household Contacts in a High Tuberculosis-Burden Area: a Comparison between Transcript Signature and Interferon Gamma Release Assay
Source: Microbiol Spectr. 2022 Apr 13;10(2):e02445-21. doi: 10.1128/spectrum.02445-21 (PMC9045189; doi:10.1128/spectrum.02445-21)
Supplement: SUPPLEMENTAL FILE 1 — Supplemental material. Download spectrum.02445-21-s001.pdf, PDF file, 0.3 MB [file spectrum.02445-21-s001.pdf]

Supplementary file

Latent Tuberculosis Infection (LTBI) diagnosis among household contacts (HHCs) in a high tuberculosis-burden area: A comparison between transcript signature and Interferon Gamma Release Assay (IGRA)

Sheetal Kaul, Vivek Nair, Shweta Birla, Shikha Dhawan, Sumit Rathore, Vishal Khanna, Sheelu Lohiya, Shakir Ali, Shamim Mannan, Kirankumar Rade, Pawan Malhotra, Dinesh Gupta, Ashwani Khanna, Asif Mohmmed

List of tables

Supplementary Table S1. qRT-PCR genes and primer sequences

Supplementary Table S2. Statistical significance for age and gender

Supplementary Table S3. ROC statistics of expression patterns of different gene combinations

Supplementary Table S4. ROC statistics of expression patterns of FCGR1B, GBP1 and GBP5 gene combination

List of figures

Supplementary Figure S1. ROC graphs of different gene combinations

Supplementary Figure S2. ROC graphs of FCGR1B, GBP1 and GBP5 gene combination

S. no.	Gene	Primer	Sequence (5'-3')	Amplicon Size
1	TNFRSF10C	Forward	TTCCTGCACCATGACCAGAG	122
		Reverse	TACTGACTTGGACTTCCCCAC	
2	ASUN	Forward	GCACGTCCTTAGCAGTTCTC	188
		Reverse	TTGCTCGTTCCAAAGGGACC	
3	NEMF	Forward	GCTTGCTAGGAATGAGAGTAAAC	196
		Reverse	CGACTCTTCAAATGTTTTCGGC	
4	FCGR1B	Forward	GGAAGGGGTGCACCGGAAGG	98
		Reverse	CACGGGGAGCAAGTGGGCAG	
5	GBP5	Forward	TGGCAAATCCTACCTGATGA	97
		Reverse	CCATATCCAAATTCCCTTGG	
6	GBP1	Forward	TGCAGGAAATGCAAAGAAAG	109
		Reverse	AACTGGACCCTGTCGTTCTC	
7	GAPDH	Forward	CAACAGCGACACCCACTCCT	115
		Reverse	CACCCTGTTGCTGTAGCCAAA	
		Reverse	GCTGATCCACATCTGCTGGAA	

Table S1. List of genes and sequences of primers used in quantitative RT-PCR

	IGRA +ve (43)	IGRA –ve (37)
Age (Mean)	31.88 (±11.10)	31.86 (±13.28)
p value for age between groups	*p*=0.995	
Female: Male	21:22	17:20
p value for gender between groups	*p*=0.796	

Table S2. Statistical significance for age and gender between IGRA +ve (Latent infection) and IGRA-ve (Uninfected) cohorts

Combination	AUC	Lower Limit	Upper Limit	z	p-value
FCGR1B, GBP1, GBP5	0.6803752	0.5570187	0.8037316	2.865913	0.00415809
GBP1, GBP5	0.6645022	0.5400303	0.788974	2.590291	0.009589485
NEMF, GBP1, GBP5, FCGR1B	0.6796537	0.5564651	0.8028423	2.858338	0.004258658
GBP1, FCGR1B	0.6567460	0.5333535	0.7801385	2.489751	0.01278327

Table S3. ROC statistics of expression patterns of different gene combinations to assess their potential to discriminate LTBI and uninfected cohorts

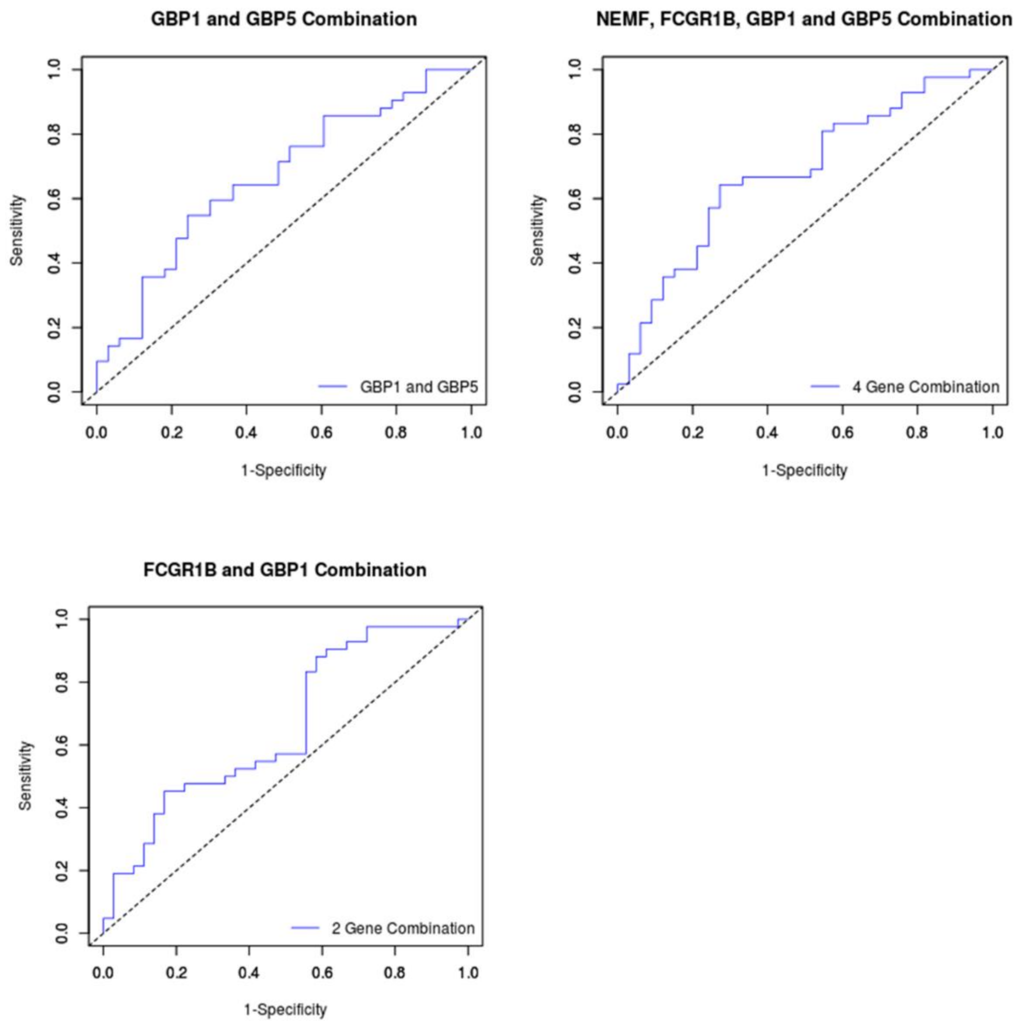

Figure S1. ROC analysis of expression patterns of different gene combinations to assess their potential to discriminate LTBI and uninfected cohorts.

FCGR1B, GBP1 and GBP5 Combination	AUC	Lower Limit	Upper Limit	z	p-value
Males	0.6666667	0.4959048	0.8374285	1.912961	0.05575308
Females	0.6966667	0.5171599	0.8761734	2.147326	0.03176733

Table S4. ROC statistics of expression patterns of FCGR1B, GBP1 and GBP5 gene combination in males and females to assess their potential to discriminate LTBI and uninfected cohorts.

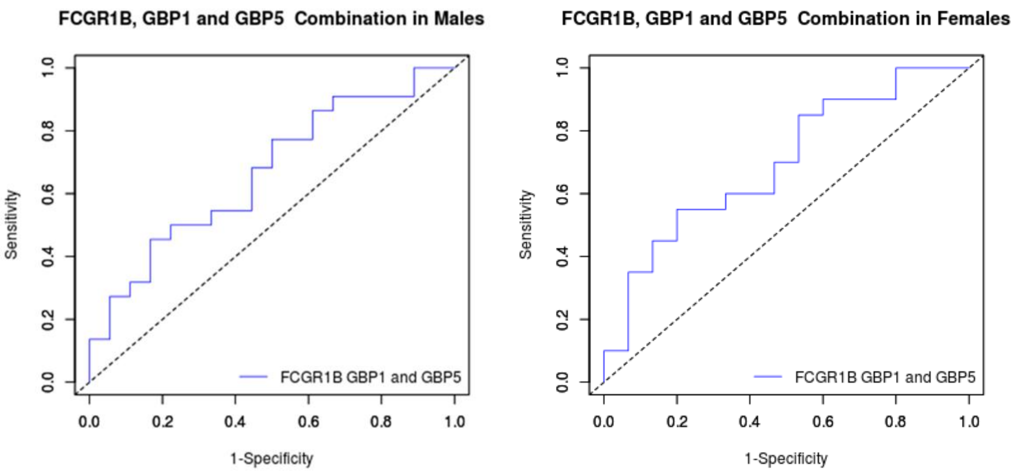

Figure S2. ROC analysis of expression patterns of FCGR1B, GBP1 and GBP5 gene combination in males and females to assess their potential to discriminate LTBI and uninfected cohorts.
